# Peritoneal Fluid Reduces Angiogenesis-Related MicroRNA Expression in Cell Cultures of Endometrial and Endometriotic Tissues from Women with Endometriosis

**DOI:** 10.1371/journal.pone.0062370

**Published:** 2013-04-19

**Authors:** Aitana Braza-Boïls, Juan Gilabert-Estellés, Luis A. Ramón, Juan Gilabert, Josep Marí-Alexandre, Melitina Chirivella, Francisco España, Amparo Estellés

**Affiliations:** 1 Grupo de Hemostasia, Trombosis, Arteriosclerosis y Biología Vascular, Instituto Investigación Sanitaria Hospital La Fe, Valencia, Spain; 2 Área Clínica Maternoinfantil, Hospital General Universitario, Valencia, Spain; 3 Servicio de Ginecología, Hospital Arnau de Vilanova, Valencia, Spain; 4 Departamento de Anatomopatología, Hospital Universitario y Politécnico La Fe, Valencia, Spain; 5 Centro de Investigación, Hospital Universitario y Politécnico La Fe, Valencia, Spain; Baylor College of Medicine, United States of America

## Abstract

Endometriosis, defined as the presence of endometrium outside the uterus, is one of the most frequent gynecological diseases. It has been suggested that modifications of both endometrial and peritoneal factors could be implicated in this disease. Endometriosis is a multifactorial disease in which angiogenesis and proteolysis are dysregulated. MicroRNAs (miRNAs) are small non-coding RNAs that regulate the protein expression and may be the main regulators of angiogenesis. Our ***hypothesis*** is that peritoneal fluid from women with endometriosis could modify the expression of several miRNAs that regulate angiogenesis and proteolysis in the endometriosis development. The ***objective*** of this study has been to evaluate the influence of endometriotic peritoneal fluid on the expression of six miRNAs related to angiogenesis, as well as several angiogenic and proteolytic factors in endometrial and endometriotic cell cultures from women with endometriosis compared with women without endometriosis.

**Methods:**

Endometrial and endometriotic cells were cultured and treated with endometriotic and control peritoneal fluid pools. We have studied the expression of six miRNAs (miR-16, -17-5p, -20a, -125a, -221, and -222) by RT-PCR and protein and mRNA levels of vascular endothelial growth factor-A, thrombospondin-1, urokinase plasminogen activator and plasminogen activator inhibitor-1 by ELISA and qRT-PCR respectively.

**Results:**

Control and endometriotic peritoneal fluid pools induced a significant reduction of all miRNAs levels in endometrial and endometriotic cell cultures. Moreover, both peritoneal fluids induced a significant increase in VEGF-A, uPA and PAI-1 protein levels in all cell cultures without significant increase in mRNA levels. Endometrial cell cultures from patients treated with endometriotic peritoneal fluid showed lower expression of miRNAs and higher expression of VEGF-A protein levels than cultures from controls. In ***conclusion***, this “in vitro” study indicates that peritoneal fluid from women with endometriosis modulates the expression of miRNAs that could contribute to the angiogenic and proteolytic disequilibrium observed in this disease.

## Introduction

Endometriosis, which is defined by the presence of endometrial tissue outside of the uterine cavity, is one of the most common gynaecologic disorders causing pelvic pain and infertility [1]–[3]. Despite the high prevalence and incapacitating symptoms of endometriosis, the precise pathogenic mechanisms of this condition remain unsolved. Sampson’s theory of retrograde menstruation is, by far, the most widely accepted [4]. However, although this theory explains the migration of endometrial fragments to ectopic locations, additional steps are required for the development of endometriotic implants [5]. The establishment of these lesions is accompanied by inflammation, neoangiogenesis and subsequent fibrosis, accounting for the symptoms described [6].

Endometriosis is a multifactorial disease in which endometrial and peritoneal factors such as those related to angiogenesis and proteolysis may be involved [7]–[9]. According to that, components of peritoneal fluid have arisen as an important field of study, provided that ectopic lesions located in the pelvic peritoneum are completely submerged in this fluid [10]–[13]. Moreover, previous studies have reported that the peritoneal fluid from women with endometriosis induces cell proliferation *in vitro*, although the mechanism underlying this effect has not further been investigated and remains unknown [13]–[14].

Angiogenesis may play an important role in the pathogenesis of endometriosis. Endometriotic implants require neovascularization to proliferate, invade the extracellular matrix, and establish an endometriotic lesion, similar to tumour metastases [6]–[7], [15]–[17]. Several studies, including ours, have reported an increase in vascular endothelial growth factor (VEGF-A) levels in endometriosis, which has been suggested to be an important angiogenic factor playing a major role in the progression of the disease [18]–[23]. Thrombospondin-1 (TSP-1), an inhibitor of angiogenesis, may also be involved in pathologies of the female reproductive tract such as endometriosis, in which vessel formation occurs [23]–[25].

Moreover, we have observed an increase in VEGF-A levels, and proteolytic factors, like urokinase plasminogen activator (uPA) and metalloproteinase-3 (MMP-3), in peritoneal fluid from patients with endometriosis in comparison with women without the disease [9], [23]. These results suggest that both tissue factors of the endometrium from women with endometriosis as well as peritoneal factors enhance the angiogenic and proteolytic capability of ectopic tissue to facilitate the implantation process.

According to previous reports, microRNAs (miRNAs) may be the main regulators of angiogenesis [26]–[30]. miRNAs are 21–22 nucleotide non-coding RNAs that regulate gene expression and play fundamental roles in biological processes. These small molecules bind to target mRNAs and mediate translational repression and/or mRNA degradation [31]. Functional analysis of miRNAs has revealed their significant regulatory influence on the expression of target genes involved in both physiological and pathological conditions [32]–[36]. Aberrant miRNA expression is associated with human diseases such as cancer, cardiovascular disorders, inflammatory diseases, and gynaecological diseases [37]–[39]. Emerging data have described a different molecular environment [40]–[41] and an altered miRNA expression in pathologic endometrium in comparison with normal endometrium [32], [42]–[45].

As we have previously described [46], VEGF-A protein levels were significantly higher in eutopic endometrium from patients than in endometrium from control women. In addition, when different endometriotic lesions were compared, VEGF-A protein levels were significantly upregulated in peritoneal lesions in comparison with ovarian or rectovaginal endometriotic lesions. In this same study miRs -16, -17-5p, -20a, -125a, -221 and -222 were quantified in different endometriotic lesions and it was demonstrated that different lesions expressed different levels of these miRNAs. Therefore, we selected these six miRNAs due to their distinct expression in different endometriotic lesions and due to “in silico” studies suggesting that these miRNAs regulate not only VEGF-A expression but other angiogenic factors [32], [44], [47]–[50].

Primary cell cultures are a useful tool for studying the response of stromal cells to peritoneal fluid components under controlled conditions. We previously studied the influence of control and endometriotic peritoneal fluid on angiogenic and proteolytic systems in endometrial cell cultures from women with and without the disease [9]. However, the effect of peritoneal fluid on angiogenesis-related miRNA expression and its correlation with angiogenesis and proteolysis in endometrial and endometriotic cell cultures have not been previously studied.

Our hypothesis is that peritoneal fluid components from women with endometriosis may alter miRNA expression in stromal cells from endometrial fragments migrated to peritoneum. Moreover, this altered miRNA expression could contribute to changes of angiogenic and proteolytic components in endometrial tissue and play a role in the establishment of the endometriotic implants.

Therefore, the aim of the present study was to investigate the effect of endometriotic peritoneal fluid on angiogenesis-related miRNA expression in endometrial cell cultures from women with endometriosis compared to control endometrial and endometriotic cell cultures and to assess whether peritoneal fluid modifies the expression of angiogenic and proteolytic factors by miRNA action.

## Materials and Methods

### Ethics Statement

Written informed consent was obtained from all patients and controls, and the study was approved by the Ethical Committee from Hospital Universitario y Politécnico La Fe, Valencia, Spain (#2008/0111).

### Clinical Groups

#### Patients

Caucasian women with moderate or severe endometriosis (stages III-IV, revised ASRM classification system, 1997) [51] were studied. Complete excision of the ovarian endometrioma (endometriotic tissue) was performed. The diagnosis of endometriosis was confirmed by anatomopathological examination of all specimens obtained. Endometrial biopsies (patient endometrial tissue) from women with moderate or severe endometriosis were performed using a cornier device without curettage, which takes the functional layer.

#### Controls

Normal endometrial tissues were obtained from fertile women without endometriosis who underwent surgery for tubal sterilization (75%) and pelvic pain (25%). The absence of endometriosis was confirmed by meticulous examination of the pelvic and extrapelvic peritoneum, ovaries, intestine, and diaphragm to detect typical or atypical endometriotic lesions. Biopsies of potential areas of endometriosis were confirmed to be negative in these women. Other gynaecologic pathologies such as adhesions or ovarian or uterine masses were also confirmed to be negative in this control group by preoperative gynaecologic ultrasound and systematic laparoscopic examination of the abdominal cavity during the intervention.

Both peritoneal fluids, from control and patients, were collected immediately after the establishment of the pneumoperitoneum and before laparoscopic manipulation. The peritoneal fluids were centrifuged at 1,500×*g* for 30 min at 4°C, filtered through a 0.2-µm pore size membrane, and stored at −80°C.

Women affected by menorrhagia or hypermenorrhea or women who had been pregnant or breast feeding during the previous 6 months were excluded from the study. None of the women had received any form of hormone therapy for at least 3 months before the study.

### Tissue Samples

Endometrial tissue (patient endometrial tissue) from 11 women with moderate or severe endometriosis (stages III-IV) (mean age 32.4 years; range 19–40), ovarian endometrioma (endometriotic tissue) from 11 women with moderate or severe endometriosis (stages III-IV) (mean age 30.5 years; range 19–42) and control endometrial tissue from 8 women without the disease (mean age 36.1 years; range 24–43) were obtained for stromal cell isolation.

No statistical significant differences in the age of the groups were observed (patient endometrial *vs* control endometrial tissue, *P* = 0.312; endometriotic tissue *vs* control endometrial tissue, *P* = 0.116).

### Peritoneal Fluid Pools

10 peritoneal fluids from women with endometriosis (endometriotic peritoneal fluid pool) (mean age 33.1 years, range 27–39) and 10 peritoneal fluids from fertile women without endometriosis (control peritoneal fluid pool) (mean age 37.2, range 21–47) in the proliferative phase of the menstrual cycle were thawed and pooled.

### Primary Cell Culture of Stromal Cells from Endometrial and Endometriotic Tissues

Primary cultures of endometrial cells were prepared as previously described [52] with minor modifications [9]. Endometrial biopsies were collected in PBS containing 50 U penicillin/mL and 50 µg streptomycin/mL (Sigma) and rinsed to remove blood cells, stored at 4°C and processed within 2–18 h. No significant correlations (*P*>0.05) between the sample processing times and the studied parameters were observed in the different groups.

Tissues were cut into 1 mm^3^ pieces and incubated at 37°C for 60 minutes in the presence of collagenase (2.5 mg/mL, Sigma). Dissociated tissues were filtered through a nylon sieve to remove undigested material. Purity of the endometrial stromal cells was higher than 95%, as evaluated by positive cellular staining for vimentin (stromal and epithelial cells) and negative cellular staining for cytokeratin (epithelial cells) and CD68 (macrophages), as previously described [9], [51]. Cell suspension was centrifuged at 550×g for 5 minutes and the pellet resuspended in DMEM-F12 phenol red-free medium containing 10% fetal bovine serum (FBS, Invitrogen), 50 U penicillin/mL and 50 µg streptomycin/mL (Sigma). Cell viability, assessed by trypan blue exclusion test, was higher than 95%. Then, stromal cells were plated and maintained in a humidified atmosphere of 5% CO_2_ and 95% air at 37°C. The culture medium was renewed every 2 days until the cell monolayer reached confluence, and then cells were subcultured. Cells were detached using 0.25% trypsin/0.02% EDTA (Gibco BRL, Paisley, UK) at 37°C and seeded at a density of 270000 cells/well in 12-well plates. Confluent cell cultures in passage 2 were used for experimental assays.

### Exposure to Peritoneal Fluid Pools

As previously described [9], preconditioning experiments to optimize the concentration of peritoneal fluid to add to endometrial cell cultures were performed. Peritoneal fluid up to 25% final concentration had no effect on cell viability, whereas higher concentrations presented toxicity. When endometrial cells in culture were treated with 0%, 10% and 25% peritoneal fluid in serum-free medium, VEGF and uPA levels augmented with increasing peritoneal fluid concentration [9]. Thereafter, endometrial cells were treated with 25% peritoneal fluid for 4 hours in all subsequent experiments.

Cells were pre-conditioned for 4 hours in FBS-free medium, shifted to FBS-free medium (ØPF) or supplemented with 25% of peritoneal fluid pools from controls (CPF) and patients (EPF), and incubated for 4 additional hours. Subsequently, cell culture supernatants were collected and aliquoted on ice, and stored at −80°C for protein quantification. RNA was obtained from cell culture extracts and stored at −80°C until use. All experiments were performed in triplicate.

To evaluate the influence of peritoneal fluid pool treatments on the expression of all studied parameters, we calculated the difference between the levels obtained with and without peritoneal fluid exposition of cell cultures from the same patient samples. Then, we correlated results from miRNAs levels with angiogenic and proteolytic factors to determine the relationship among these parameters.

### RNA Extraction

Total RNA from primary cell cultures were extracted using the mirVana miRNA isolation kit (Ambion, USA), according to the manufacturer’s protocol. Yield and purity of RNA were measured using a NanoDrop ND-1000 spectrophotometer (Nanodrop Technologies).

### Quantification of mRNAs

One microgram of total RNA was treated with DNase I (Invitrogen, Carlsbad, CA) and reverse transcribed into first-strand cDNA by using Superscript RNase H^-^ (Invitrogen) with an oligo (dT)_15_ primer (Promega, USA). cDNA was stored at −20°C until subsequent study. Analysis of VEGF-A, TSP-1, uPA, PAI-1 and β-actin mRNA expression was performed in a Light Cycler termocycler, using version 3.5 software (Roche Molecular Biochemicals, Germany). The specific primers used for amplification of VEGF-A, TSP-1 and β-actin, the reaction mixture, and the PCR conditions were performed as previously described [43].

### Quantification of Mature miRNAs

For this study we selected a set of six miRNAs involved in angiogenesis (miR-16, -17-5p, -20a, -125a, -221 and -222) and RNU6B (small nuclear RNA) as endogenous control.

Quantification using the TaqMan MicroRNA Assays was done using two-step RT-PCR in total RNA samples. This method quantifies exclusively mature miRNAs but not their precursors. In the reverse transcription, cDNA is reverse transcribed from total RNA samples using specific miRNA primers from the TaqMan MicroRNA Assays and reagents from the TaqMan® MicroRNA Reverse Transcription Kit (Applied Biosystems, Foster City CA). Reactions were performed in an Eppendorf Mastercycler Thermocycler following the manufacture’s protocol. Then, PCR products were amplified from cDNA samples using the Taqman MicroRNA Assay and the TaqMan® Universal PCR Master Mix (Applied Biosystems, USA).

Relative quantification of miRNA expression was calculated with the 2^−ΔΔCt^ method (Applied Biosystems user bulletin no. 2), using RNU6B as endogenous control. Data are presented as fold change relative to the mean of control endometrial cell culture without peritoneal fluid exposure (control endometrial culture = 1).

### Determination of Angiogenic and Proteolytic Factors

VEGF-A protein level was measured using a commercially available ELISA (Human VEGF, IBL International, Germany). No cross-reactivity or interference with platelet derived growth factor was observed. This assay recognizes human VEGF-A_165_ and VEGF-A_121_ isoforms. The intra-assay and inter-assay variation coefficients were 4–6% and 7–10%, respectively.

TSP-1 protein level was quantified using a commercially available ELISA (Human TSP-1, ELISA Development System, DuoSet, RD systems, USA). No cross-reactivity or interference with TSP-2 or TSP-4 was observed. The intra-assay and inter-assay variation coefficients were 5–6% and 8–11%, respectively.

uPA protein levels were quantified by a commercially available ELISA (Zymutest uPA, Hyphen Biomed, France), which measures single-chain urokinase (scuPA) and the high weight molecular form of uPA (HMW-uPA) with similar efficiency. The intra-assay and inter-assay variation coefficients were 3–5% and 8–11%, respectively.

PAI-1 protein levels were quantified by a commercially available ELISA (Imubind tissue PAI-1, America Diagnostica, USA). The assay detects free and complexed PAI-1 and is insensitive to PAI-2. The intra-assay and inter-assay variation coefficients were 3–4% and 6–8%, respectively.

VEGF, uPA, TSP1 and PAI-1 protein levels were determined in culture supernatants and in peritoneal fluid pools. The protein amounts released to the culture medium by cells incubated with peritoneal fluid pools were calculated by subtracting VEGF, TSP1, uPA and PAI-1 contents in the peritoneal fluid pool to the total levels obtained in supernatants.

### Statistical Analysis

All the studied parameters showed a normal distribution. Values were expressed as mean ± standard error of the mean (SEM). Differences between means were analyzed by one-way ANOVA test. When significant *P* values were observed, post-hoc analyses were performed using Bonferroni test. Differences between studied variables in the endometrial cells from women with endometriosis vs women without endometriosis for the same treatment were analyzed by unpaired Student t-test. Correlations between variables were calculated by the bivariate Pearson correlation test. *P* values <0.05 (two-tailed) were considered significant. Statistical tests were performed using the statistical package SPSS Release 20 for Windows (SPSS Inc.).

## Results

**Figure 1:**
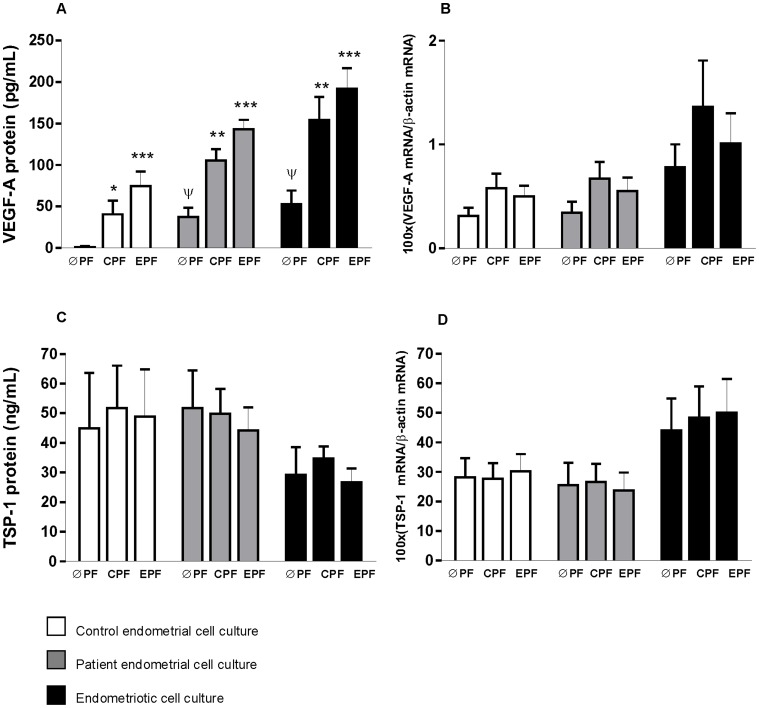
Peritoneal fluid (PF) effects on VEGF-A and TSP-1 expression in stromal cell cultures from control endometrial tissue, and patient endometrial and endometriotic tissues from women with endometriosis. ØPF, without PF; CPF, control PF; EPF, endometriotic PF. Data are expressed as Mean ± SEM. * *p*<0.05; ** *p*<0.01; *** *p*<0.001; vs ØPF from same tissue culture, ψ *p*<0.05 vs ØPF from control endometrium culture. A: VEGF-A protein; B: VEGF-A mRNA; C: TSP-1 protein; D: TSP-1 mRNA.

**Figure 2:**
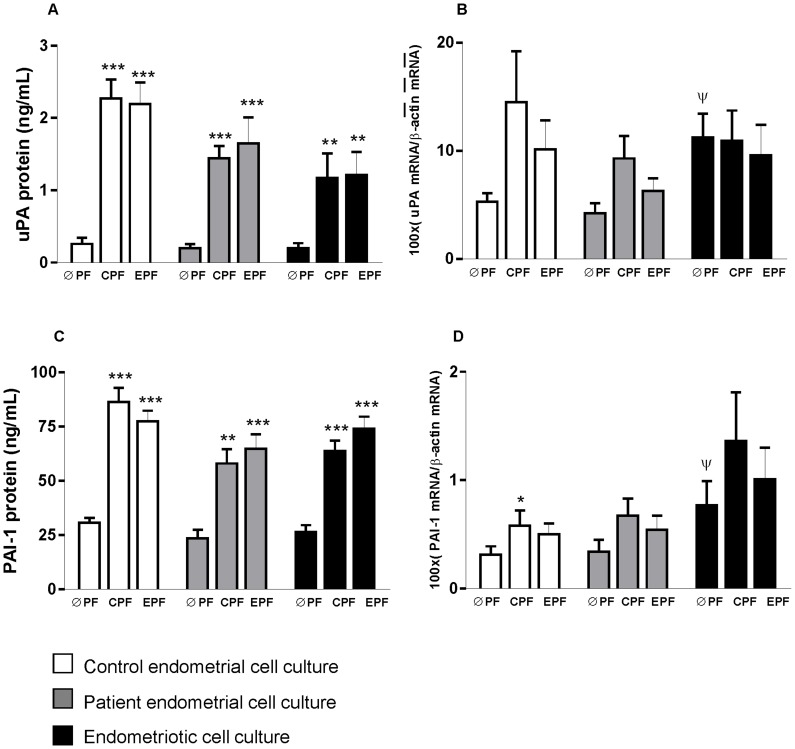
Peritoneal fluid (PF) effects on uPA and PAI-1 expression in stromal cell cultures from control endometrial tissue, and patient endometrial and endometriotic tissues from women with endometriosis. ØPF, without PF; CPF, control PF; EPF, endometriotic PF. Data are expressed as Mean ± SEM. * *p*<0.05; ** *p*<0.01; *** *p*<0.001; vs ØPF from same tissue culture, ψ *p*<0.05 vs ØPF from control endometrium culture. A: uPA protein; B: uPA mRNA; C: PAI-1 protein; D: PAI-1 mRNA.

**Figure 3:**
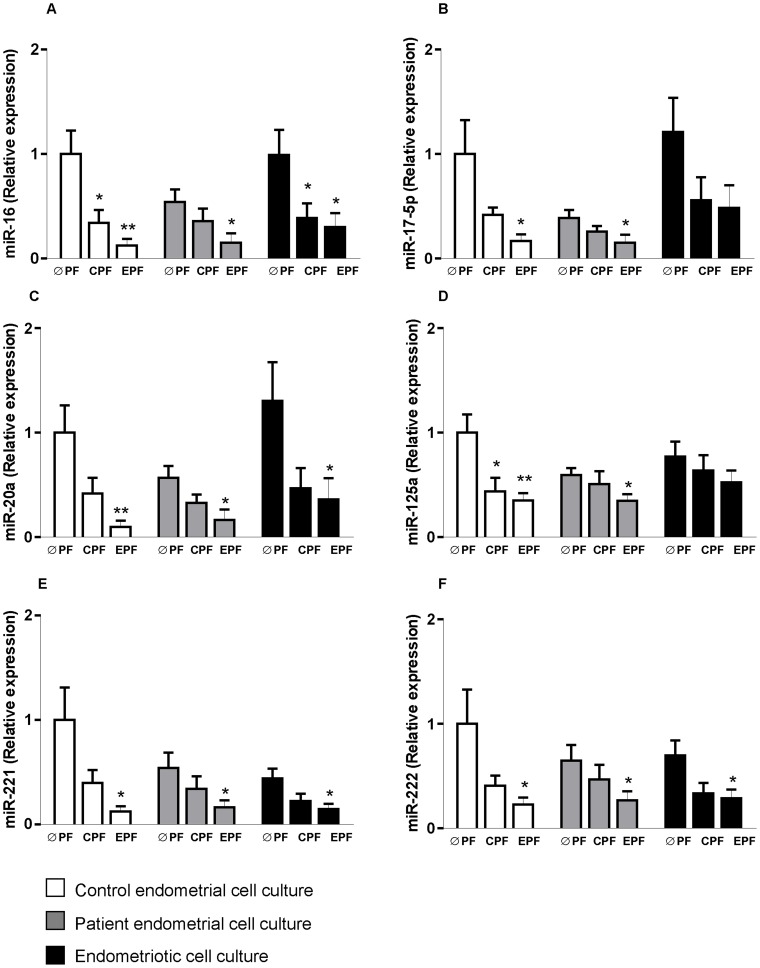
Peritoneal fluid (PF) effects on miRNA expression in stromal cell cultures from control endometrial tissue, and patient endometrial and endometriotic tissues from women with endometriosis. ØPF, without PF; CPF, control PF; EPF, endometriotic PF. *p<0.05; **p<0.01; *p*<0.001 vs ØPF same tissue culture. miRNA expression is presented as fold change relative to control stromal cell culture without peritoneal fluid (control endometrium ØPF = 1). A: miR-16; B: miR-17-5p; C: miR-20a; D: miR-125a; E: miR-221; F: miR-222.

**Figure 4:**
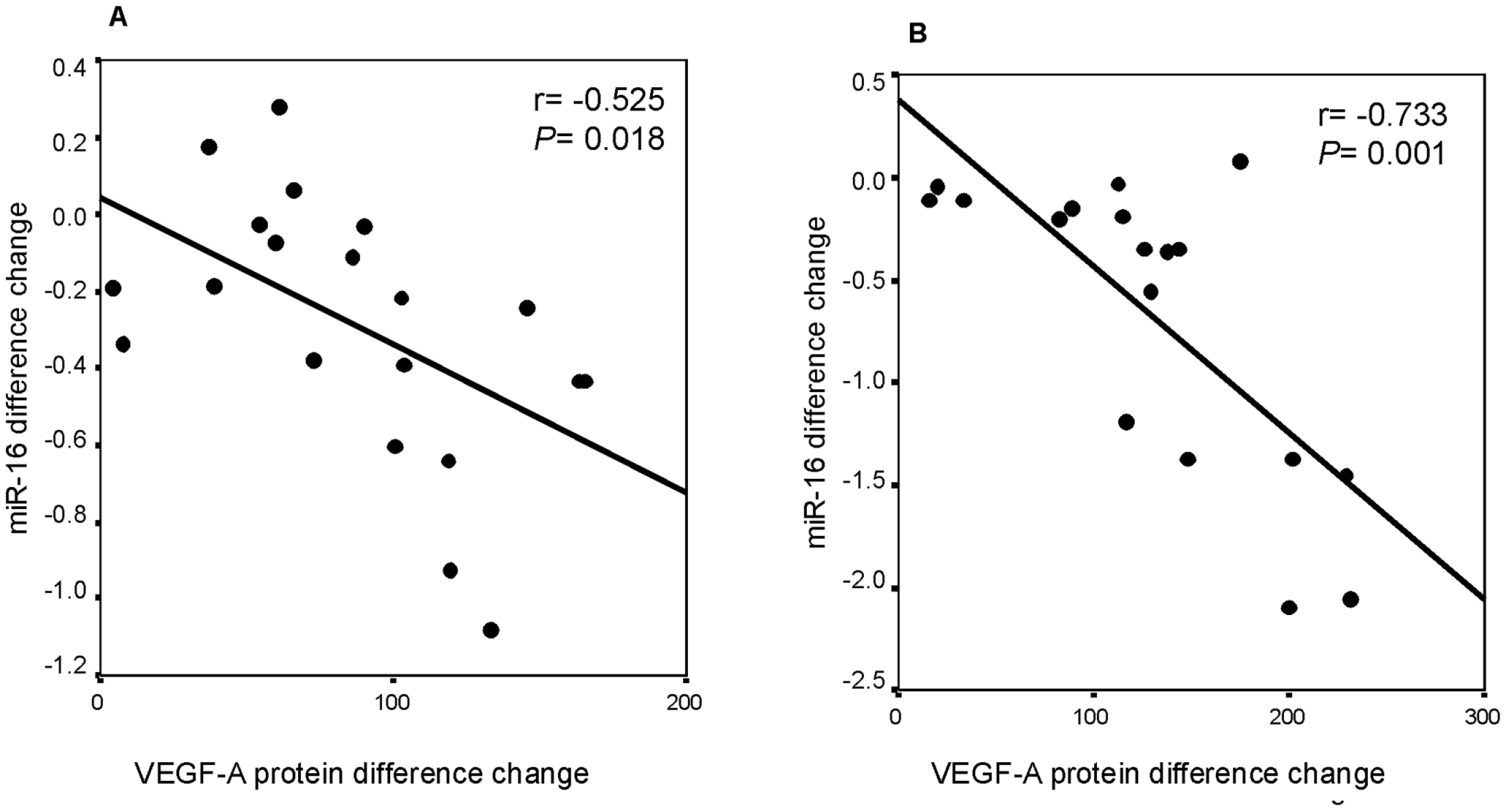
Correlation between changes in miR-16 vs. VEGF-A protein levels in eutopic endometrial (A) and endometriotic cell cultures (B) from women with endometriosis after treatment with both peritoneal fluid pools compared to cell cultures without peritoneal fluid treatment. Change: differences between parameter levels with and without peritoneal fluid treatment.

Confluent cultures of stromal cells from patient and control endometrial tissues and endometriotic tissues were treated with serum-free medium containing control or endometriotic peritoneal fluid pools (25% final concentration) or without peritoneal fluid pools (0% final concentration) for 4 hours. Figures 1–4 show levels of different parameters measured in all experimental conditions.

### Effect of Peritoneal Fluid on VEGF-A and TSP-1 Levels of Endometrial Cell Cultures from Patient and Control Endometrial Tissues and Endometriotic Tissues (Figure 1)

Control and endometriotic peritoneal fluids significantly enhanced VEGF-A protein levels in all tissue cultures when compared with the corresponding cell culture without peritoneal fluid (Fig 1A). However, peritoneal fluid pools did not significantly modify mRNA expression of VEGF-A (Fig. 1B). The highest VEGF-A protein level was observed in endometrial and endometriotic cell cultures from women with endometriosis treated with the endometriotic peritoneal fluid pool (Fig.1A).

In addition to VEGF-A, which is the most important inductor of angiogenesis, we also studied the main inhibitor of angiogenesis, TSP-1. Treatment of endometrial and endometriotic cell cultures with peritoneal fluid did not significantly modify TSP-1 expression (Fig 1C and 1D).

In the absence of peritoneal fluid exposure, a significant increase (*P*<0.05) in VEGF-A protein levels was observed in endometriotic cell cultures (52.90±16.45 pg/ml) and patient endometrial cell cultures (37.14±11.22 pg/ml) in comparison with control endometrial cell cultures (1.23±0.07 pg/ml) (Fig 1 A).

### Effect of Peritoneal Fluid on uPA and PAI-1 Levels of Endometrial Cell Cultures from Patient and Control Endometrial Tissues and Endometriotic Tissues (Figure 2)

Control and endometriotic peritoneal fluid pools induced a similar significant increase in uPA and PAI-1 protein expression (Fig 2A, Fig 2C) without significantly modifying mRNA levels in primary cell cultures of endometrial and endometriotic stromal cells (Fig 2B, Fig 2D).

### Effect of Peritoneal Fluid on the Level of miRNAs Related to Angiogenesis (Figure 3)

We selected 6 miRNAs that could be involved in the regulation of angiogenic factors (miR-16, -17-5p, -20a, -125a, -221 and –222).

Exposure of control endometrial cells to peritoneal fluid (Figure 3, white bars), reduced the level of the six miRNAs studied. Exposure of patient endometrial tissue (grey bars) and endometriotic tissue (black bars) cells to peritoneal fluid also reduced miRNA levels, but this reduction was not always statistically significant. The highest miRNA decrease was observed after exposure to endometriotic peritoneal fluid in all cases. Results are expressed as fold change relative to the mean of cell cultures from control endometrium without peritoneal fluid treatments.

### Correlation between Changes in miRNA Expression and Changes in Angiogenic and Fibrinolytic Parameters after Treatment with Peritoneal Fluid Pools

To evaluate the influence of peritoneal fluid on the response of the angiogenic system we calculated the difference between each treatment and the basal expression of each studied parameter. We correlated the difference observed in angiogenic or proteolytic factors with the difference observed in miRNA expression after peritoneal fluid pool treatments.

A significant inverse correlation was observed between changes in VEGF-A protein and miR-16 levels in eutopic endometrial and endometriotic cell cultures from women with endometriosis after treatment with peritoneal fluid (r = −0.525, *P* = 0.018 and r = −0.733, *P* = 0.001, respectively) (Figure 4). Furthermore, a significant inverse correlation was observed between changes in VEGF-A protein and miR-17-5p *(*r = −0.739, *P* = 0.001), miR -20a *(*r = −0.676, *P* = 0.001), miR-125a (r = −0.567, *P* = 0.01) and miR-222 (r = −0.494, *P* = 0.037) levels in endometriotic cell cultures from women with endometriosis after treatment with peritoneal fluid.

Finally, assessment of the changes in expression of angiogenic and proteolytic factors in response to peritoneal fluid revealed a significant positive correlation between VEGF-A and uPA levels in control endometrial cultures with and without peritoneal fluid exposure (r = 0.719, *P* = 0.001).

## Discussion

This study evaluates the influence of peritoneal fluid from women with or without endometriosis on the expression of six miRNAs that modulate angiogenesis, as well as several angiogenic and proteolytic factors, in endometriotic and endometrial cell cultures from women with and without endometriosis.

Several studies have indicated that endometrium and peritoneal fluid from women with endometriosis have different expression patterns of several angiogenic and proteolytic components in comparison with endometrium and peritoneal fluid from control women, suggesting that these systems play a role in the pathogenesis of endometriosis [18]–[21], [23], [24], [53]–[55]. Peritoneal fluid is a dynamic media with continuous changes in the volume, the cellular components and cytokines. Moreover, it has been described that some inflammatory, immunological and oxidative stress components are dysregulated in endometriotic peritoneal fluid [56]–[60]. All of these alterations could dysregulate miRNA expression in stromal cells of endometrial fragments migrated to peritoneum, facilitating the implantation of ectopic lesions. In a previous report, we showed that endometrial-peritoneal interactions increased the expression of angiogenic and proteolytic factors in endometrial cells and suggested that this contributes to the establishment of endometriotic lesions [9]. However, the specific mechanisms by which peritoneal fluid components modulate the expression of angiogenic and proteolytic factors in endometrial and endometriotic cells have not been previously elucidated.

The present study shows that peritoneal fluid from women with endometriosis induces the highest decrease in angiogenesis-related miRNAs and the highest increase in VEGF-A protein levels in endometrial cell cultures from patients. The increase in protein levels without significant modification of mRNA levels could suggest a miRNA-mediated action on post-transcriptional regulation.

miRNAs, which have emerged as important regulators of gene expression, are involved in most cellular processes and many diseases, including endometriosis [44]–[46], [61], [62]. An increasing number of studies have described the relationship between miRNAs and angiogenesis [26]–[28], [47] and emerging data suggest that dysregulation of miRNA expression is involved in endometriosis [32], [35], [43], [45], [46], increasing the likelihood that miRNAs could be used as biomarkers and therapeutic tools for this disease [38], [63], [64].

In the present study, treatment with peritoneal fluid resulted in decreased levels of miRNAs related to angiogenesis (miR-16, -17-5p, -20a, -125a, -221 and –222) and an increase in VEGF-A protein levels. The six miRNAs assessed in this study were selected for this *in vitro* model because they regulate VEGF-A expression directly or indirectly [28], [29], [44], [65]. We found a significant correlation between the decrease in miR-16 and the increase in VEGF-A in response to peritoneal fluid exposure in endometrial and endometriotic cell cultures. This correlation could indicate regulation of VEGF-A translation by miR-16. It has been shown that VEGF-A is a target gene for miR-16 in several cell types, indicating that miR-16 could be an important regulator of angiogenesis [30], [47], [46]. However, further experiments would be needed to test the hypothesis.

Furthermore, a significant inverse correlation was observed after peritoneal fluid treatment between the increase in VEGF-A protein expression and miR-17-5p, miR-20a, miR-125a and miR-222 levels in endometriotic cell cultures. These results suggest that peritoneal fluid modulates angiogenesis in endometrial and endometriotic stromal cells via miRNA action.

miRNAs -17-5p and -20a are contained in the miR-17-92 cluster, which has a complex role in angiogenesis [30], [66], [67]. While miR-17-5p, has pro-angiogenic activity [68], [69], miR-20a displays anti-angiogenic activity by targeting VEGF-A [70]. Recently, Doebele *et al.* [66] showed that miR-17 and miR-20a exhibit a cell-intrinsic anti-angiogenic activity in endothelial cells. Another angiomiRs, miR-221 and miR-222, with a demonstrated anti-angiogenic activity by targeting c-kit, have been shown to inhibit endothelial cell migration, proliferation, and angiogenesis *in vitro* [71]–[73].

Several groups have studied peritoneal-endometrial interactions, showing that peritoneal fluid components (cytokines, growth factors, steroid hormones, and angiogenic and proteolytic factors) play an important role in the pathogenesis of endometriosis [13], [56], [57], [74]. Moreover, cell types such as macrophages and endometrial and red blood cells have been detected in peritoneal fluid. Endometriotic peritoneal fluid reportedly induces the production and release of VEGF by neutrophils [9], [12], [54]. Our data show that endometriotic peritoneal fluid induced the highest increase in VEGF-A protein levels and the lowest miRNA expression in endometrial cell culture from women with endometriosis. Therefore, we hypothesized that an increase in cytokines or growth factors present in endometriotic peritoneal fluid contribute to the increase in angiogenic processes via a reduction of miRNAs that inhibit the translation of angiogenic factors. Hence, this reduction would induce an increase in protein levels of their target genes.

Extracellular remodelling is necessary during the initial stages of angiogenesis. The enzymes involved in extracellular proteolysis include components of the plasminogen system [75], [76]. Given that VEGF-A has been shown to induce uPA expression [75], it seemed reasonable to study angiogenic and fibrinolytic systems in our study subjects simultaneously. Furthermore, it has been reported that uPA levels are significantly higher in the endometrium from women with endometriosis than in controls [23], [53], [55], [77]–[79], In the present study, we observed a significant induction of uPA and PAI-1 protein in the stromal cell culture in response to the presence of both peritoneal fluids. Moreover, a significant positive correlation was observed between the changes in VEGF-A and uPA protein levels in control endometrial culture after exposure to peritoneal fluid pools. The increase in proteolytic factors induced by peritoneal fluid from patients may also favour angiogenesis and invasive properties in this tissue.

The relationship between protein and mRNA levels of several angiogenic and proteolytic components may reflect the posttranscriptional regulation of the expression of these factors and their role in the development of endometriosis. It is important to emphasize that peritoneal fluid induced the expression of VEGF-A and uPA proteins but not of their corresponding mRNAs in the present study, which could be explained by miRNA action.

Limitations of the present study: In this study we have only evaluated six miRNAs, but it would be important to enlarge the number of angiomiRs to assess the importance of miRNAs in the regulation of the angiogenesis in the endometriosis development. Future functional studies (gain and loss of function experiments) are required to confirm the correlations between miRNAs and protein levels observed in the present study. Endometriosis is a multifactorial disease in which systems like angiogenesis, inflammation, immune system or epigenetics may be implicated in the pathogenesis of this disease. So, studying miRNAs action on establishment of ectopic lesions it is not enough to complain this complicated disease. It would be necessary to evaluate the implication of other systems in miRNAs regulation to better understand the cellular processes under the endometriosis development.

## Conclusions

In this study we have observed that peritoneal fluid from women with endometriosis decreased the expression of six angiomiRs that could act as modulators of the translation of angiogenic and proteolytic factors in patient endometrial cells. Moreover, we have observed an inverse significative correlation between miRNA and protein levels. However, further validation of the targets of these differentially expressed miRNAs is necessary to clarify the role of miRNAs in the regulation of angiogenesis in endometriosis.

## References

[1] Burney RO, Giudice LC (2012) Pathogenesis and pathophysiology of endometriosis. Fertil Steril 98: 511–9. 22819144 10.1016/j.fertnstert.2012.06.029 PMC3836682

[2] Giudice LC, Kao LC (2004) Endometriosis. Lancet 364: 1789–1799. 15541453 10.1016/S0140-6736(04)17403-5

[3] Bulun SE (2009) Endometriosis. N Engl J Med 360: 268–279. 19144942 10.1056/NEJMra0804690

[4] Sampson JA (1927) Peritoneal endometriosis due to menstrual dissemination of endometrial tissues into the peritoneal cavity. Am J Obstet Gynecol 14: 422–469.

[5] Hapangama DK, Raju RS, Valentijn AJ, Barraclough D, Hart A, et al (2012) Aberrant expression of metastasis-inducing proteins in ectopic and matched eutopic endometrium of women with endometriosis: implications for the pathogenesis of endometriosis. Hum Reprod 27: 394–407. 22147918 10.1093/humrep/der412

[6] Laschke MW, Menger MD (2007) In vitro and in vivo approaches to study angiogenesis in the pathophysiology and therapy of endometriosis. Hum Reprod Update 13: 331–342. 17347159 10.1093/humupd/dmm006

[7] Kobayashi H (2000) Invasive capacity of heterotopic endometrium. Gynecol Obstet Invest 50 Suppl: 26–32 11093058 10.1159/000052875

[8] Zondervan KT, Treloar SA, Lin J, Weeks DE, Nyholt DR, et al (2007) Significant evidence of one or more susceptibility loci for endometriosis with near-Mendelian inheritance on chromosome 7p13–15. Hum Reprod 22: 717–728. 17158817 10.1093/humrep/del446

[9] Cosín R, Gilabert-Estellés J, Ramón LA, Gómez-Lechón MJ, Gilabert J, et al (2010) Influence of peritoneal fluid on the expression of angiogenic and proteolytic factors in cultures of endometrial cells from women with endometriosis. Hum Reprod 25: 398–405. 19945964 10.1093/humrep/dep419

[10] Koninckx PR, Kennedy SH, Barlow DH (1998) Endometriotic disease: the role of peritoneal fluid. Hum Reprod Update 4: 741–751. 10027629 10.1093/humupd/4.5.741

[11] Mier-Cabrera J, Jiménez-Zamudio L, García-Latorre E, Cruz-Orozco O, Hernández-Guerrero C (2011) Quantitative and qualitative peritoneal immune profiles, T-cell apoptosis and oxidative stress-associated characteristics in women with minimal and mild endometriosis. BJOG 118: 6–16. 10.1111/j.1471-0528.2010.02777.x 21083865

[12] Na YJ, Lee DH, Kim SC, Joo JK, Wang JW, et al (2010) Effects of peritoneal fluid from endometriosis patients on the release of monocyte-specific chemokines by leukocytes. Arch Gynecol Obstet 283: 1333–1341. 20617440 10.1007/s00404-010-1583-1

[13] Liu Y, Hu J, Shen W, Wang J, Chen C, et al (2011) Peritoneal fluid of patients with endometriosis promotes proliferation of endometrial stromal cells and induces COX-2 expression. Fertil Steril 95: 1836–1838. 21145050 10.1016/j.fertnstert.2010.11.039

[14] Minici F, Tiberi F, Tropea A, Orlando M, Gangale MF, et al (2008) Endometriosis and human infertility: a new investigation into the role of eutopic endometrium. Hum Reprod 23: 530–537. 18096563 10.1093/humrep/dem399

[15] McLaren J (2000) Vascular endothelial growth factor and endometriotic angiogenesis. Hum Reprod Update 6: 45–55. 10711829 10.1093/humupd/6.1.45

[16] Laschke MW, Elitzsch A, Vollmar B, Vajkoczy P, Menger MD (2006) Combined inhibition of vascular endothelial growth factor (VEGF), fibroblast growth factor and platelet-derived growth factor, but not inhibition of VEGF alone, effectively suppresses angiogenesis and vessel maturation in endometriotic lesions. Hum Reprod 21: 262–268. 16172144 10.1093/humrep/dei308

[17] Laschke MW, Giebels C, Menger MD (2011) Vasculogenesis: a new piece of the endometriosis puzzle. Hum Reprod Update 17: 628–636. 21586449 10.1093/humupd/dmr023

[18] Donnez J, Smoes P, Gillerot S, Casanas-Roux F, Nisolle M (1998) Vascular endothelial growth factor (VEGF) in endometriosis. Hum Reprod 13: 1686–1690. 9688413 10.1093/humrep/13.6.1686

[19] Fasciani A, D’Ambrogio G, Bocci G, Monti M, Genazzani AR, et al (2000) High concentrations of the vascular endothelial growth factor and interleukin-8 in ovarian endometrioma. Mol Hum Reprod 6: 50–54. 10611260 10.1093/molehr/6.1.50

[20] McLaren J, Prentice A, Charnock-Jones DS, Smith SK (1996) Vascular endothelial growth factor (VEGF) concentrations are elevated in peritoneal fluid of women with endometriosis. Hum Reprod 11: 220–223. 8671190 10.1093/oxfordjournals.humrep.a019023

[21] Takehara M, Ueda M, Yamashita Y, Terai Y, Hung YC, et al (2004) Vascular endothelial growth factor A and C gene expression in endometriosis. Hum Pathol 35: 1369–1375. 15668894 10.1016/j.humpath.2004.07.020

[22] Girling JE, Rogers PA (2005) Recent advances in endometrial angiogenesis research. Angiogenesis 8: 89–99. 16211359 10.1007/s10456-005-9006-9

[23] Gilabert-Estellés J, Ramón LA, España F, Gilabert J, Vila V, et al (2007) Expression of angiogenic factors in endometriosis: its relation to fibrinolytic and metalloproteinase (MMP) systems. Hum Reprod 22: 2120–2127. 17609243 10.1093/humrep/dem149

[24] Tan XJ, Lang JH, Liu DY, Shen K, Leng JH, et al (2002) Expression of vascular endothelial growth factor and thrombospondin-1 mRNA in patients with endometriosis. Fertil Steril 78: 148–153. 12095505 10.1016/s0015-0282(02)03187-4

[25] Kawano Y, Nakamura S, Nasu K, Fukuda J, Narahara H, et al (2005) Expression and regulation of thrombospondin-1 by human endometrial stromal cells. Fertil Steril 83: 1056–1059. 15820829 10.1016/j.fertnstert.2004.09.035

[26] Fish JE, Srivastava D (2009) MicroRNAs: opening a new vein in angiogenesis research. Sci Signal 2: pe1 doi: 10.1126/scisignal.252pe1 19126861 10.1126/scisignal.252pe1 PMC2680274

[27] Suarez Y, Sessa WC (2009) MicroRNAs as novel regulators of angiogenesis. Circ Res 104: 442–454. 19246688 10.1161/CIRCRESAHA.108.191270 PMC2760389

[28] Wu F, Yang Z, Li G (2009) Role of specific microRNAs for endothelial function and angiogenesis. Biochem Biophys Res Commun 386: 549–553. 19540203 10.1016/j.bbrc.2009.06.075 PMC2821898

[29] Wang S, Olson EN (2009) AngiomiRs-key regulators of angiogenesis. Curr Opin Genet Dev 19: 205–211. 19446450 10.1016/j.gde.2009.04.002 PMC2696563

[30] Caporali A, Emanueli C (2011) MicroRNA regulation in angiogenesis. Vascul Pharmacol 55: 79–86. 21777698 10.1016/j.vph.2011.06.006

[31] Bartel DP (2004) MicroRNAs: genomics, biogenesis, mechanism, and function. Cell 116: 281–297. 14744438 10.1016/s0092-8674(04)00045-5

[32] Ohlsson-Teague EM, Van der Hoek KH, Van der Hoek MB, Perry N, Wagaarachchi P, et al (2009) MicroRNA-regulated pathways associated with endometriosis. Mol Endocrinol 23: 265–275. 19074548 10.1210/me.2008-0387 PMC5419313

[33] Ambros V (2004) The functions of animal microRNAs. Nature 16 431: 350–355. 10.1038/nature02871 15372042

[34] Burney RO, Hamilton AE, Aghajanova L, Vo KC, Nezhat CN, et al (2009) MicroRNA expression profiling of eutopic secretory endometrium in women with versus without endometriosis. Mol Hum Reprod 15: 625–631. 19692421 10.1093/molehr/gap068 PMC2744474

[35] Kuokkanen S, Chen B, Ojalvo L, Benard L, Santoro N, et al (2010) Genomic profiling of microRNAs and messenger RNAs reveals hormonal regulation in microRNA expression in human endometrium. Biol Reprod 82: 791–801. 19864316 10.1095/biolreprod.109.081059 PMC2842492

[36] Ramón LA, Braza-Boïls A, Gilabert J, España F, Chirivella M, et al (2012) microRNAs related to angiogenesis are dysregulated in endometriod endometrial cancer. Human Reprod 27: 3036–3045. 10.1093/humrep/des292 22904162

[37] Sonkoly E, Pivarcsi A (2009) microRNAs in inflammation. Int Rev Immunol 28: 535–561. 19954362 10.3109/08830180903208303

[38] Wittmann J, Jäck HM (2010) Serum microRNAs as powerful cancer biomarkers. Biochim Biophys Acta 1806: 200–207. 20637263 10.1016/j.bbcan.2010.07.002

[39] Gilabert-Estellés J, Braza-Boïls A, Ramón LA, Zorio E, Medina P, et al (2012) Role of microRNAs in gynecological pathology. Curr Med Chem 19: 2406–2413. 22455593 10.2174/092986712800269362

[40] Guo SW (2009) Epigenetics of endometriosis. Mol Hum Reprod 15: 587–607. 19651637 10.1093/molehr/gap064

[41] Aghajanova L, Giudice LC (2011) Molecular evidence for differences in endometrium in severe versus mild endometriosis. Reprod Sci 18: 229–251. 21063030 10.1177/1933719110386241 PMC3118406

[42] Pan Q, Luo X, Toloubeydokhti T, Chegini N (2007) The expression profile of micro-RNA in endometrium and endometriosis and the influence of ovarian steroids on their expression. Mol Hum Reprod 13: 797–806. 17766684 10.1093/molehr/gam063

[43] Pan Q, Chegini N (2008) MicroRNA signature and regulatory functions in the endometrium during normal and disease states. Semin Reprod Med 26: 479–493. 18951330 10.1055/s-0028-1096128 PMC2728121

[44] Ohlsson-Teague EM, Print CG, Hull ML (2010) The role of microRNAs in endometriosis and associated reproductive conditions. Hum Reprod Update 16: 146–165. 10.1093/humupd/dmp034 19773286

[45] Hawkins SM, Creighton CJ, Han DY, Zariff A, Anderson ML, et al (2011) Functional microRNA involved in endometriosis. Mol Endocrinol 25: 821–832. 21436257 10.1210/me.2010-0371 PMC3082329

[46] Ramón LA, Braza-Boïls A, Gilabert-Estellés J, Gilabert J, España F, et al (2011) microRNAs expression in endometriosis: its relation to angiogenic factors. Human Reprod 26: 1082–1090. 10.1093/humrep/der025 21335415

[47] Urbich C, Kuehbacher A, Dimmeler S (2008) Role of microRNAs in vascular diseases, inflammation, and angiogenesis. Cardiovasc Res 79: 581–588. 18550634 10.1093/cvr/cvn156

[48] Chamorro-Jorganes A, Araldi E, Penalva LO, Sandhu D, Fernández-Hernando C, et al (2011) MicroRNA-16 and microRNA-424 regulate cell-autonomous angiogenic functions in endothelial cells via targeting vascular endothelial growth factor receptor-2 and fibroblast growth factor receptor-1. ArteriosclerThromb Vasc Biol 31: 2595–2606. 10.1161/ATVBAHA.111.236521 PMC3226744 21885851

[49] Yang W, Lee DY, Ben-David Y (2011) The roles of microRNAs in tumorigenesis and angiogenesis. Int J Physiol Pathophysiol Pharmacol 3: 140–155. 21760972 PMC3134008

[50] Patella F, Rainaldi G (2011) MicroRNAs mediate metabolic stresses and angiogenesis. Cell Mol Life Sci 69: 1049–1065. 21842412 10.1007/s00018-011-0775-6 PMC11115142

[51] American Society for Reproductive Medicine (1997) Revised American Society for Reproductive Medicine Classification of endometriosis. Fertil Steril 67: 817–821. 9130884 10.1016/s0015-0282(97)81391-x

[52] Hirota Y, Osuga Y, Koga K, Yoshino O, Hirata T, et al (2005) Possible implication of midkine in the development of endometriosis. Hum Reprod 20: 1084–1089. 15734764 10.1093/humrep/deh720

[53] Ramón L, Gilabert-Estellés J, Castelló R, Gilabert J, España F, et al (2005) mRNA analysis of several components of the plasminogen activator and matrix metalloproteinase systems in endometriosis using a real-time quantitative RT-PCR assay. Hum Reprod 20: 272–278. 15579491 10.1093/humrep/deh571

[54] Gilabert-Estellés J, Estellés A, Gilabert J, Castelló R, España F, et al (2003) Expression of several components of the plasminogen activator and matrix metalloproteinase systems in endometriosis. Hum Reprod 18: 1516–1522. 12832381 10.1093/humrep/deg300

[55] Cho S, Choi YS, Jeon YE, Im KJ, Choi YM, et al (2012) Expression of vascular endothelial growth factor (VEGF) and its soluble receptor-1 in endometriosis. Microvasc Res 83: 237–242. 22230112 10.1016/j.mvr.2011.12.004

[56] Velasco I, Acién P, Campos A, Acién MI, Ruiz-Maciá E (2010) Interleukin-6 and other soluble factors in peritoneal fluid and endometriomas and their relation to pain and aromatase expression. J Reprod Immunol 84: 199–205. 20074813 10.1016/j.jri.2009.11.004

[57] Michaud N, Al-Akoum M, Gagnon G, Girard K, Blanchet P, et al (2011) Decreased concentrations of soluble interleukin-1 receptor accessory protein levels in the peritoneal fluid of women with endometriosis. J Reprod Immunol 92: 68–73. 21958553 10.1016/j.jri.2011.08.001

[58] McKinnon B, Bersinger NA, Wotzkow C, Mueller MD (2012) Endometriosis-associated nerve fibers, peritoneal fluid cytokine concentrations, and pain in endometriotic lesions from different locations. Fertil Steril 97: 373–380. 22154765 10.1016/j.fertnstert.2011.11.011

[59] Drosdzol-Cop A, Skrzypulec-Plinta V, Stojko R (2012) Serum and peritoneal fluid immunological markers in adolescent girls with chronic pelvic pain. Obstet Gynecol Surv 67: 374–381. 22713164 10.1097/OGX.0b013e31825cb12b

[60] Carvalho LF, Abrão MS, Biscotti C, Sharma R, Nutter B, et al. (2013) Oxidative Cell Injury as a Predictor of Endometriosis Progression. Reprod Sci. doi: 10.1177/1933719112466301. 10.1177/1933719112466301 23287096

[61] Ng EK, Wong CL, Ma ES, Kwong A (2009) MicroRNAs as new Players for diagnosis, prognosis, and therapeutic targets in breast cancer. J Oncol 2009: 1–6. 10.1155/2009/305420 PMC2716485 19644558

[62] Zorio E, Medina P, Rueda J, Millán JM, Arnau MA, et al (2009) Insights of the role of microRNAs in cardiac diseases: from biological signaling to therapeutic targets. Cardiovas Hematol Agent Med Chem 7: 82–90. 10.2174/187152509787047676 19149547

[63] Gilad S, Meiri E, Yogev Y, Benjamin S, Lebanony D, et al (2008) Serum microRNAs are promising novel biomarkers. PLoS One 3: e3148. 18773077 10.1371/journal.pone.0003148 PMC2519789

[64] Reid G, Kirschner MB, van Zandwijk N (2011) Circulating microRNAs: Association with disease and potential use as biomarkers. Crit Rev Oncol Hematol 80: 193–208. 21145252 10.1016/j.critrevonc.2010.11.004

[65] Qin B, Yang H, Xiao B (2012) Role of microRNAs in endothelial inflammation and senescence. Mol Biol Rep 39: 4509–4518. 21952822 10.1007/s11033-011-1241-0

[66] Doebele C, Bonauer A, Fischer A, Scholz A, Reiss Y, et al (2010) Members of the microRNA-17–92 cluster exhibit a cell-intrinsic antiangiogenic function in endothelial cells. Blood 115: 4944–4950. 20299512 10.1182/blood-2010-01-264812

[67] Mendell JT (2008) miRiad roles for the miR-17–92 cluster in development and disease. Cell 133: 217–322. 18423194 10.1016/j.cell.2008.04.001 PMC2732113

[68] Dews M, Homayouni A, Yu D, Murphy D, Sevignani C, et al (2006) Augmentation of tumor angiogenesis by a Myc-activated microRNA cluster. Nat Genet 38: 1060–1065. 16878133 10.1038/ng1855 PMC2669546

[69] Suárez Y, Fernández-Hernando C, Yu J, Gerber SA, Harrison KD, et al (2008) Dicer-dependent endothelial microRNAs are necessary for postnatal angiogenesis. Proc Natl Acad Sci U S A 105: 14082–14087. 18779589 10.1073/pnas.0804597105 PMC2544582

[70] Hua Z, Lv Q, Ye W, Wong CK, Cai G, et al (2006) MiRNA-directed regulation of VEGF and other angiogenic factors under hypoxia. PLoS One 1: e116. 17205120 10.1371/journal.pone.0000116 PMC1762435

[71] Poliseno L, Tuccoli A, Mariani L, Evangelista M, Citti L, et al (2006) MicroRNAs modulate the angiogenic properties of HUVECs. Blood 108: 3068–3071. 16849646 10.1182/blood-2006-01-012369

[72] Wu F, Yang Z, Li G (2009) Role of specific microRNAs for endothelial function and angiogenesis. Biochem Biophys Res Commun 386: 549–553. 19540203 10.1016/j.bbrc.2009.06.075 PMC2821898

[73] Li Y, Song YH, Li F, Yang T, Lu YW, et al (2009) MicroRNA-221 regulates high glucose-induced endothelial dysfunction. Biochem Biophys Res Commun 381: 81–83. 19351599 10.1016/j.bbrc.2009.02.013 PMC2670889

[74] Sacco K, Portelli M, Pollacco J, Schembri-Wismayer P, Calleja-Agius J (2012) The role of prostaglandin E(2) in endometriosis. Gynecol Endocrinol 28: 134–138. 22003899 10.3109/09513590.2011.588753

[75] Pepper MS (2001) Role of the matrix metalloproteinase and plasminogen activator-plasmin systems in angiogenesis. Arterioscler Thromb Vasc Biol 21: 1104–1107. 11451738 10.1161/hq0701.093685

[76] Zorio E, Gilabert-Estellés J, España F, Ramón LA, Cosín R, et al (2008) Fibrinolysis: the key to new pathogenetic mechanisms. Curr Med Chem 15: 923–929. 18473800 10.2174/092986708783955455

[77] Gilabert-Estelles J, Castello R, Gilabert J, Ramon LA, España F, et al (2005) Plasminogen activators and plasminogen activator inhibitors in endometriosis. Front Biosci 10: 1162–1176. 15769615 10.2741/1609

[78] Osteen KG, Bruner KL, Sharpe-Timms KL (1996) Steroid and growth factor regulation of matrix metalloproteinase expression and endometriosis. Semin Reprod Endocrinol 14: 247–255. 8885055 10.1055/s-2007-1016334

[79] Sillem M, Prifti S, Koch A, Neher M, Jauckus J, et al (2001) Regulation of matrix metalloproteinases and their inhibitors in uterine endometrial cells of patients with and without endometriosis. Eur J Obstet Gynecol Reprod Biol 95: 167–174. 11301163 10.1016/s0301-2115(00)00415-2

